# Zr-89 Immuno-PET Targeting Ectopic ATP Synthase Enables In-Vivo Imaging of Tumor Angiogenesis

**DOI:** 10.3390/ijms20163928

**Published:** 2019-08-13

**Authors:** Bok-Nam Park, Ga-Hee Kim, Seung-A Ko, Ga-Hee Shin, Su-Jin Lee, Young-Sil An, Joon-Kee Yoon

**Affiliations:** Department of Nuclear Medicine & Molecular Imaging, Ajou University School of Medicine, Worldcup-ro 164, Suwon 16499, Korea

**Keywords:** immuno-PET, Zr-89, ATP synthase, angiogenesis

## Abstract

In this study, we synthesized a Zr-89-labeled anti-adenosine triphosphate synthase monoclonal antibody (ATPS mAb) for applications in immuno-positron emission tomography (PET) and evaluated its feasibility for angiogenesis imaging. The cellular uptake of Zr-89 ATPS mAb was measured after treatment of cancer cell lines in vitro, and its biodistribution was evaluated at 4, 24 and 48 h in vivo in mice bearing xenografts. PET images were acquired at 4, 24, 48, and 96 h after Zr-89 ATPS mAb administration. Tumor angiogenesis was analyzed using anti-CD31 immunofluorescence staining. The cellular uptake of Zr-89 ATPS mAb increased over time in MDA-MB-231 breast cancer cells but did not increase in PC3 prostate cancer cells. The tumor uptake of Zr-89 ATPS mAb at 24 h was 9.4 ± 0.9% ID/g for MDA-Mb-231 cells and was 3.8 ± 0.6% ID/g for PC3 cells (*p =* 0.004). Zr-89 ATPS mAb uptake in MDA-MB-231 xenografts was inhibited by the administration of cold ATPS mAb (4.4 ± 0.5% ID/g, *p =* 0.011). Zr-89 ATPS mAb uptake could be visualized by PET for up to 96 h in MDA-MB-231 tumors. In contrast, there was no distinct tumor uptake detected by PET in the PC3 xenograft model. CD31-positive tumor vessels were abundant in MDA-MB-231 tumors, whereas they were scarcely detected in PC3 tumors. In conclusion, ATPS mAb was successfully labeled with Zr-89, which could be used for immuno-PET imaging targeting tumor angiogenesis.

## 1. Introduction

Angiogenesis, the development of new capillary blood vessels, is crucial for the growth and metastasis of tumors, and has thus been widely investigated as a target for tumor imaging and therapy. Angiostatin is an endogenous anti-angiogenic factor that suppresses the growth of tumors at remote sites by maintaining the local angiogenic balance against proangiogenic factors such as vascular endothelial growth factor (VEGF) and fibroblast growth factor [1]. Adenosine triphosphate synthase (ATPS) comprises two functional domains: F_1_ and F_O_. Normally, ATPS localizes in the cytoplasm; however, it is also observed at the plasma membranes of endothelial cells and tumor cells. The α/β subunits of this protein serve as the binding sites for angiostatin [2]. Angiostatin, along with antibodies against the α and β subunits, have been shown to inhibit the activity of ATPS on the endothelial cell surface [3,4]. Therefore, ATPS is a marker for tumor angiogenesis as well as a target for anti-angiogenesis therapy. 

Positron emission tomography (PET) is a clinically proven technology for cancer imaging, especially for staging/restaging, detection of recurrence, and prognostic prediction [5]. Recently, radiopharmaceuticals other than F-18 fluorodeoxyglucose (FDG) have been thoroughly investigated. Immuno-PET is a rapidly growing discipline, which combines the high sensitivity and accurate quantification of PET with the specific targeting of monoclonal antibodies (mAbs) [6]. In contrast to commonly used PET radioisotopes with relatively short half-lives, such as F-18 (t_1/2_ = 110 min) and C-11 (t_1/2_ = 20 min), which do not attune with the in-vivo circulation time of intact antibodies, long-lived radioisotopes such as I-124 (t_1/2_ = 4.18 days) and Zr-89 (t_1/2_ = 3.27 days) enable prolonged imaging of the mAb distribution using PET [7]. To date, hundreds of preclinical and clinical studies have been performed to develop Zr-89-based immuno-PET imaging using Food and Drug Administration-approved and analytical mAbs. Human epidermal growth factor receptor 2 (HER2), prostate-specific membrane antigen (PSMA), VEGF, and CD20 are currently the most common targets of immuno-PET imaging [8].

We previously demonstrated the feasibility of using a radioiodine-labeled ATPS mAb as a theragnostic imaging agent in an in-vivo xenograft model [9]. Gastric cancer could be visualized under gamma imaging as of 24 h after administration of the I-125 labeled ATPS mAb, and the tumor-to-background ratio increased steadily as of 48 h after administration. In addition, as a radioimmunotherapeutic agent, the I-131-labeled ATPS mAb significantly inhibited the growth of gastric cancer compared to controls. 

Based on these results, in the present study, we synthesized a Zr-89-labeled ATPS mAb for immuno-PET imaging and evaluated its feasibility in angiogenesis imaging using cancer cells lines in in-vitro and in-vivo xenograft models. 

## 2. Results

### 2.1. Radiosynthesis of Zr-89 ATPS mAb 

**Figure 1:**
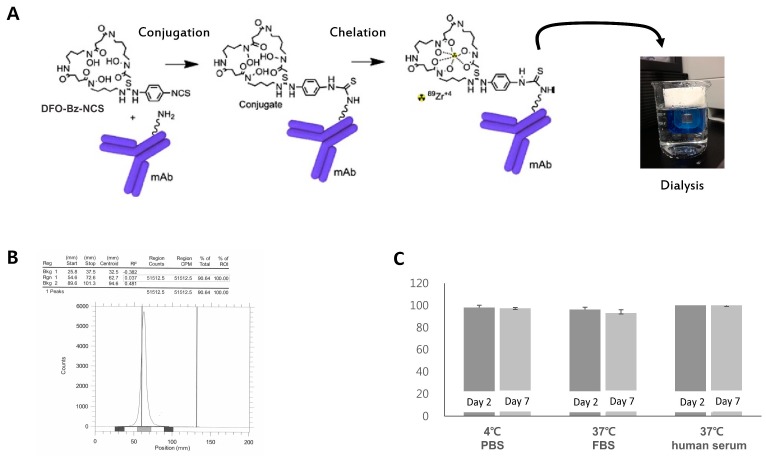
Schematic diagram for radiosynthesis of Zr-89 ATPS mAb (**A**). DF-Bz-NCS was first conjugated with ATPS mAb, and then Zr-89 was chelated to the conjugate. Labeling efficiency (**B**) and in-vitro stability (**C**) of Zr-89 ATPS mAb measured by radio-TLC. ATPS, Adenosine Triphosphate Synthase; mAb, Monoclonal antibody; Df-Bz-NCS, p-isothiocyanatobenzyl-desferrioxamine. Error bar = standard error.

The Zr-89 ATPS mAb was successfully synthesized according to the schematic representation shown in Figure 1A. The radiochemical yield of Zr-89 ATPS mAb was 100.0% (Figure 1B). The retention factors ranged from 0.0 to 0.1 for Zr-89 ATPS mAb and from 0.9 to 1.0 for free Zr-89. The in-vitro stability of Zr-89 ATPS mAb in phosphate-buffered saline (PBS) at 4 °C was 98.2 ± 1.8% and 97.3 ± 0.8% on the 2nd and 7th days of radiolabeling, respectively, with no statistically significant difference (*p =* 0.753, Figure 1C). Similarly, the in-vitro stability of the Zr-89 ATPS mAb in fetal bovine serum (FBS) at 37 °C was not significantly altered between the 2nd day (96.3 ± 2.0%) and the 7th day (93.1 ± 2.9%, *p =* 0.577). The in-vitro stability in human serum at 37 °C reached 100 ± 0.0% both on the 2nd and 7th days. 

### 2.2. Western Blotting and Confocal Microscopy for ATPS Expression

Western blot analysis with ATPS mAb displayed a single protein band at 52 kDa in six cancer cell lines (Figure S1A). The intensity of the ATPS protein band was higher in FTC133, HT1080, and MDA-MB-231 cells than in MKN45, PC3, and A549 cells for total membranes. Similarly, for the plasma membranes, the protein bands in FTC133, HT1080, and MDA-MB-231 cells were prominent; however, the bands were not clearly visualized in MKN45, PC3, and A549 cells. 

Immunofluorescence microscopy with ATPS mAb was employed to detect APTS expression in the six cancer cell lines. We observed fluorescence signals with punctate appearance in FTC133, HT1080, and MDA-MB-231 cells, which suggested the ectopic expression of ATPS (Figure S1B) [3,10]. However, MKN45, PC3, and A549 cells displayed fluorescence signals with a fine granular cytoplasmic pattern throughout. Based on these results, MDA-MB-231 and PC3 cells were chosen as positive and negative controls for ectopic ATPS expression, respectively, which were used for the subsequent cellular and small animal experiments. 

### 2.3. Cell Uptake and Saturation Binding Assay

**Figure 2:**
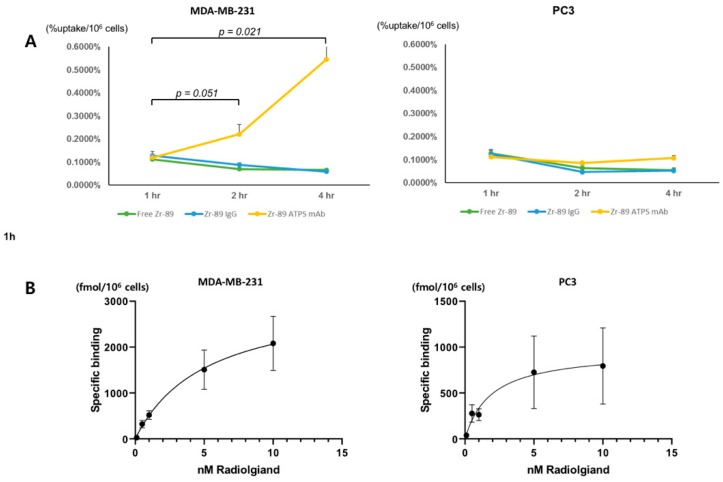
Cellular uptake of Zr-89 ATPS mAb at 1, 2, and 4 h of incubation in MDA-MB-231 and PC3 cells (**A**). The cellular uptake is presented as the percentage of the radioactivity added per 10^6^ cells. Saturation binding assay of Zr-89 ATPS mAb in MDA-MB-231 and PC3 cells (**B**). Specific binding is plotted against the radioligand concentrations. ATPS, adenosine triphosphate synthase; mAb, monoclonal antibody. Error bars = standard errors.

The cellular uptake of Zr-89 ATPS mAb was measured in MDA-MB-231 and PC3 cells and presented as the percentage of the radioactivity added per 10^6^ cells (Figure 2A). For MDA-MB-231 cells, the cellular uptake of Zr-89 ATPS mAb increased over time; 0.103 ± 0.014%/10^6^ cells, 0.266 ± 0.058%/10^6^ cells and 0.556 ± 0.122%/10^6^ cells at 1, 2 and 4 h, respectively. Cellular uptake was significantly higher at 4 h than at 1 h after incubation (*p* = 0.021). However, cellular uptake of free Zr-89 and Zr-89 IgG did not increase significantly; for free Zr-89, 0.106 ± 0.013%/10^6^ cells, 0.070 ± 0.014%/10^6^ cells and 0.072 ± 0.005%/10^6^ cells at 1, 2 and 4 h, respectively; for Zr-89 IgG, 0.108 ± 0.022%/10^6^ cells, 0.089 ± 0.005%/10^6^ cells and 0.071 ± 0.013%/10^6^ cells at 1, 2 and 4 h, respectively. In contrast, for PC3 cells, cellular uptake of all three radiotracers did not increase with time; for Zr-89 ATPS mAb, 0.112 ± 0.019%/10^6^ cells, 0.086 ± 0.005%/10^6^ cells and 0.108 ± 0.005%/10^6^ cells at 1, 2 and 4 h, respectively; for free Zr-89, 0.129 ± 0.015%/10^6^ cells, 0.064 ± 0.014%/10^6^ cells and 0.056 ± 0.009%/10^6^ cells at 1, 2 and 4 h, respectively; for Zr-89 IgG, 0.124 ± 0.019%/10^6^ cells, 0.047 ± 0.010%/10^6^ cells and 0.053 ± 0.009%/10^6^ cells at 1, 2 and 4 h, respectively. 

The saturation binding assay revealed nanomolar concentrations of K_d_ at 5.2 ± 1.7 nM for MDA-MB-231 cells and 1.9 ± 0.8 nM for PC3 cells (Figure 2B). Total numbers of receptors expressed on the cells (B_max_) were 3130 ± 547 fM/10^6^ cells for MDA-MB-231 and 962 ± 203 fM/10^6^ cells for PC3.

### 2.4. Biodistribution/Inhibition Study

**Figure 3:**
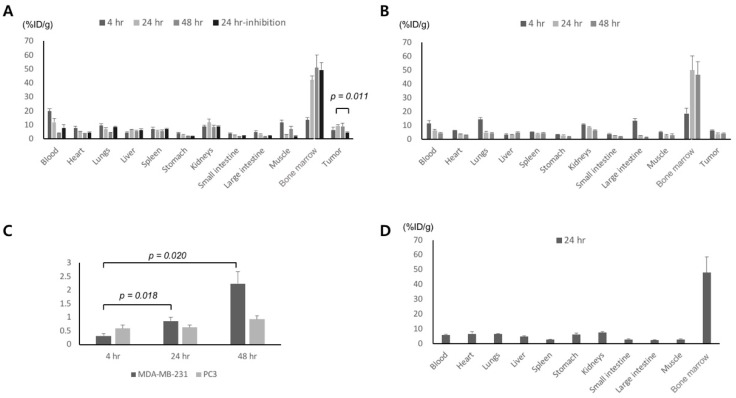
The biodistribution of Zr-89 ATPS mAb at 4, 24, and 48 h in mice bearing MDA-MB-231 tumors (**A**) and PC3 tumors (**B**). Uptake of Zr-89 ATPS mAb was inhibited by unlabeled ATPS mAb at 24 h for MDA-MB-231 tumors. Tumor -to-blood ratio of Zr-89 ATPS mAb (**C**). The biodistribution of Zr-89 oxalate at 24 h in wild-type mice (**D**). ATPS, adenosine triphosphate synthase; mAb, monoclonal antibody. Error bars = standard errors. Arrows indicate tumors.

The biodistribution of Zr-89 ATPS mAb was performed using mice bearing MDA-MB-231 tumors (Figure 3A) or PC3 tumors (Figure 3B) at 4, 24 and 48 h. Tumor uptake of Zr-89 ATPS mAb in MDA-MB-231 tumors increased with time, although it was not statistically significant at 6.5 ± 2.0% ID/g, 9.4 ± 0.9% ID/g, and 9.0 ± 2.0% ID/g at 4, 24, and 48 h, respectively. Tumor uptake of Zr-89 ATPS mAb in PC3 tumors decreased but was not statistically significant at 24 and 48 h; 6.3 ± 0.1% ID/g, 3.8 ± 0.1% ID/g, and 4.0 ± 0.5% ID/g at 4, 24, and 48 h, respectively. Tumor uptake of Zr-89 ATPS mAb at 24 h was significantly higher for MDA-MB-231 tumors than for PC3 tumors (*p* = 0.004). Zr-89 ATPS mAb uptake in MDA-MB-231 tumors at 24 h was inhibited by cold ATPS mAb (4.4 ± 0.5% ID/g, *p* = 0.011). The tumor/blood ratio of Zr-89 ATPS mAb in MDA-MB-231 tumors increased significantly with time, with 0.32 ± 0.09, 0.87 ± 0.13, and 2.24 ± 0.44 at 4, 24, and 48 h, respectively (Figure 3C, *p* < 0.05). In contrast, the tumor/blood ratio of Zr-89 ATPS mAb in PC3 tumors did not increase with time, with 0.59 ± 0.13, 0.63 ± 0.09, and 0.93 ± 0.13 observed at 4, 24, and 48 h, respectively (*p* > 0.05). Zr-89 ATPS mAb uptake in the blood was the highest at 4 h and then decreased with time, at 19.8 ± 1.9% ID/g and 4.1 ± 0.3% ID/g at 4 and 48 h, respectively, for MDA-MB-231 tumors, and 11.5 ± 2.0% ID/g and 4.5 ± 0.4% ID/g at 4 and 48 h, respectively for PC3 tumors. Similarly, Zr-89 ATPS mAb uptake in the heart and muscles also reached its peak at 4 h. However, the bone marrow uptake of Zr-89 ATPS mAb increased with time for both tumor models; 13.5 ± 2.0% ID/g, 42.2 ± 3.0% ID/g, and 51.0 ± 7.4% ID/g at 4, 24, and 48 h, respectively, for MDA-MB-231 tumors, and 18.5 ± 3.8% ID/g, 49.9 ± 10.3% ID/g, and 46.4 ± 9.5% ID/g at 4, 24, and 48 h, respectively for PC3 tumors. We also evaluated the biodistribution of Zr-89 oxalate after injection to the wild-type mice (Figure 3D). At 24 h after administration, the bone marrow uptake was 48.4 ± 10.4% ID/g, which was similar to that of Zr-89 ATPS mAb at 24 h. 

### 2.5. PET and confocal imaging

**Figure 4:**
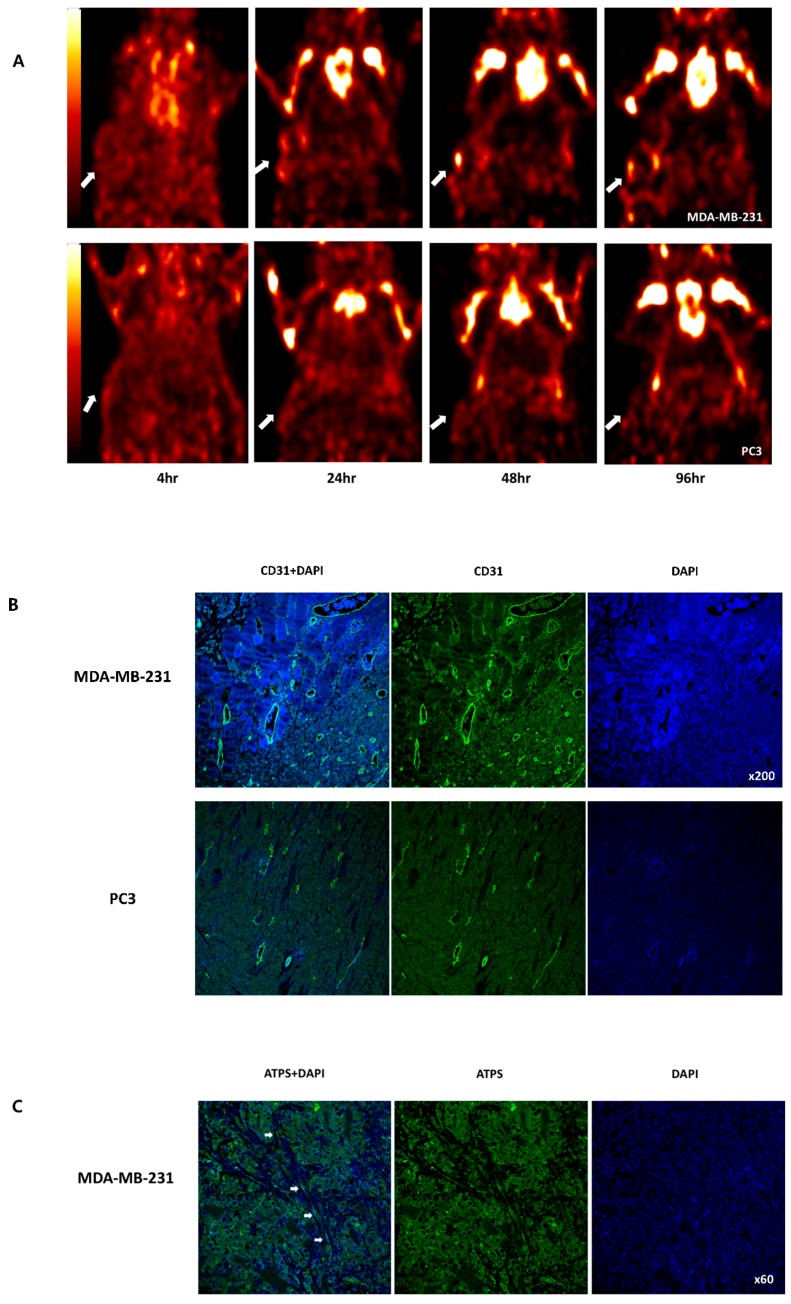
PET imaging for the biodistribution of Zr-89 ATPS mAb at 4, 24, 48, and 96 h in mice bearing MDA-MB-231 (upper) or PC3 (lower) tumors in the right flank (**A**). Confocal microscopy with anti-CD31 antibody for tumor angiogenesis imaging (**B**) and with ATPS mAb for visualizing the expression of ATPS on the endothelial cells. ATPS, adenosine triphosphate synthase; mAb, monoclonal antibody. Arrows indicate tumors (**A**) and endothelial cells (**C**).

To confirm the biodistribution results, PET images were acquired at 4, 24, 48, and 96 h after administration of Zr-89 ATPS mAb for MDA-MB-231 and PC3 tumors (Figure 4A). On PET images, Zr-89 ATPS mAb uptake in MDA-MB-231 tumors was visualized from 24 h after administration The tumor uptake was maintained until 96 h, indicating that the uptake was not due to the blood flow but rather antigen-specific uptake. In contrast, Zr-89 ATPS mAb uptake was similar to the background activity for PC3 tumors at any time point. Maximum standardized uptake values (SUVmax) were 1.61 at 24 h, 0.93 at 48 h and 1.13 at 96 h for MDA-MB-231 tumors, while those for PC3 tumors were 0.86 at 24 h, 0.51 at 48 h and 0.66 at 96 h. As expected from the biodistribution results, bone marrow uptake was observed as of 24 h and prevailed for 96 h for both tumor models. 

Confocal microscopy images were in line with PET imaging results. CD31-positive tumor vessels were abundant in MDA-MB-231 tumors, whereas they were scarcely detected in PC3 tumors (Figure 4B). Additionally, the expression of ATPS on the endothelial cells of MDA-MB-231 xenograft was visualized by confocal microscopy (Figure 4C). 

## 3. Discussion

In the present study, to develop an immuno-PET imaging agent against the tumor angiogenesis, ATPS mAb was coupled to Zr-89 by conjugation with p-isothiocyanatobenzyl-desferrioxamine B (Df-Bz-NCS). Based on the results of confocal microscopy and western blot analysis for ATPS expression, MDA-MB-231 cells were chosen as the ATPS-positive cell line, while PC3 cells served as the negative control. MDA-MB-231 cells showed a five-fold increase of Zr-89 ATPS mAb uptake after 4 h incubation in vitro. The in-vivo biodistribution analysis revealed uptake of 9.4 ± 0.9% ID/g at 24 h after administration, which was specifically inhibited by a large amount of cold ATPS mAb. On PET imaging, MDA-MB-231 tumors were visible 4 h after injection of Zr-89 ATPS mAb, which further corresponded with the level of angiogenesis evaluated by CD31 expression. By contrast, PC3 tumors showed lower mAb uptake in the biodistribution analysis, and the tumors were not visible on PET imaging. These results indicate that immuno-PET using Zr-89 ATPS mAb could be applied in imaging tumor angiogenesis. 

Zr-89 is a long-lived radioisotope suitable for immuno-PET imaging. As an isotope of a radiometal, it requires a chelator for coupling with antibodies. Ever since the first report of desferal as a chelator for Zr-89 [11], various Zr-89-labeled antibodies have been developed. Among these, Zr-89 trastuzumab is the most frequently studied. PET imaging with Zr-89 trastuzumab could be used to noninvasively assess the HER2 expression status of tumors to guide anti-HER2 therapy in breast cancer models [12]. Zr-89 trastuzumab PET also enabled monitoring the antitumor activity of a tyrosine kinase inhibitor in HER2-positive gastric cancer models, in which F-18 FDG PET failed to demonstrate the effect of treatment [13]. PSMA is another potential target for Zr-89 immuno-PET, which is currently the most promising target for prostate cancer. As F-18 FDG is not sensitive to indolent prostate cancers, several other radiopharmaceuticals have been investigated to replace F-18 FDG [14]. A human pilot study demonstrated that the tumor uptake of Zr-89-labeled J591, an mAb against PSMA, correlated with the Gleason score and was helpful for the accurate identification of index lesions [15]. 

In concordance with our previous study [9], we successfully labeled Zr-89 to ATPS mAb for angiogenesis imaging using PET. Another option for immuno-PET imaging with antibodies is I-124, which has a long half-life that is comparable to the in-vivo circulation time of intact antibodies. Compared with Zr-89, I-124 has an advantage of well-established labeling methods. However, owing to its long positron range and low γ-ray energy, the image quality of I-124 PET was shown to be inferior to that of Zr-89 PET [16]. Our previous study showed that a large amount of free radioiodine cleaved in vivo and accumulated in the thyroid glands, with uptake of 2875.5 ± 51.8% ID/g and 1916.5 ± 173.4% ID/g at 24 and 48 h after administration of I-125 ATPS mAb, respectively [9]. The use of potassium iodide for thyroid protection is mandatory when radio-iodinated ATPS mAb is applied to patients. In the present study, high bone marrow uptake (42.2–51.0% ID/g) was found at 24 and 48 h after administration of Zr-89 ATPS mAb, even though high in-vitro stability was obtained until 7 days in FBS at 37 °C. Moreover, the biodistribution data from Zr-89 oxalate demonstrated a similar degree of bone marrow uptake at 24 h after injection (48.0 ± 10.4% ID/g). These results indicate that the bone marrow uptake occurs not by deconjugation of DF-Bz-NCS from the antibody, but rather by the dechelation of Zr-89 from DF-Bz-NCS in vivo [17]. A recent report supports this finding [18]. In particular, the authors showed that the bone marrow uptake of Zr-89 chloride and Zr-89 oxalate reached up to 20% ID/g at 8 h and persisted until 6 days after administration, whereas the whole body activity of Zr-89 desferrioxamine was completely cleared after the 1st day of administration. Although the bone marrow uptake of free Zr-89 was much lower than the thyroid uptake of free radioiodine, it may hinder the detection of bone metastasis in cancer patients. Therefore, further improvements in labeling methods for more stable chelation are necessary before clinical application. 

During the course of our research, we have also made several other attempts to synthesize Zr-89-labeled antibody fragments for ATPS. Through these efforts, we obtained Fab and F(ab’)_2_ fragments by enzymatic cleavage. Unfortunately, we found that the fragments had no advantage over whole antibodies in terms of tumor uptake and biodistribution. Zr-89 ATPS Fab was rapidly cleared by the kidneys, and the tumor uptake was lower than that of Zr-89 ATPS mAb. Consequently, Zr-89 ATPS F(ab’)_2_ showed a similar biodistribution to Zr-89 ATPS mAb. There was no difference in tumor uptake between Zr-89 ATPS F(ab’)_2_ and Zr-89 ATPS mAb. Moreover, the bone marrow uptake of Zr-89 ATPS F(ab’)_2_ was equivalent to that of Zr-89 ATPS mAb at 24 and 48 h after administration (data not shown). However, considering the protein loss incurred during the fragmentation process and the additional time required for the synthesis of radiopharmaceuticals, whole antibodies are superior to antibody fragments for Zr-89-based immuno-PET imaging. 

One limitation of this study is that the γ-ray energy window was set to 250–700 keV for PET imaging. The spontaneous decay of Zr-89 emitting high-energy photons of 909 KeV with a high yield (0.99) is beyond the window range of a conventional PET system. As a result, copious amounts of scattered photons cause high background activity and reduce image quality [19]. Because this is an inherent limitation of current PET systems, other researchers have adopted energy windows similar to ours (250–400 KeV as a lower limit, 650–700 KeV as an upper limit) [20,21,22]. Nevertheless, for adequate clinical application, further technical optimization for Zr-89 PET imaging is required. 

In summary, Zr-89 ATPS mAb uptake was specific for tumor angiogenesis, which was revealed by in-vitro cellular uptake, in-vivo biodistribution, PET imaging, and anti-angiogenesis staining. The results of this study suggest that Zr-89 ATPS mAb could be used for immuno-PET imaging targeting tumor angiogenesis. Further studies to improve the in-vivo stability of Zr-89 ATPS mAb by structurally modifying the chelators and optimizing the acquisition of PET data by adjusting the window range for Zr-89 are warranted.

## 4. Materials and Methods 

### 4.1. Radiosynthesis of Zr-89-Labeled ATPS mAb 

ATPS mAb was purchased from Abcam (MW 52 kDa, Cambridge, MA, USA) and stored in aliquots at −78 °C. Zr-89 oxalate was obtained from PerkinElmer (Boston, MA, USA). For chelation, a five- to ten-fold molar excess of Df-Bz-NCS (in 20 μL dimethyl sulfoxide) was added to the ATPS mAb (100–200 μg in 10–20 μL 0.1 N NaHCO_3_ buffer, pH 9.0) with gentle shaking, and incubated for 30 min at 37 °C. Unconjugated chelate was removed using Slide-A-Lyzer™ Dialysis Cassettes (2 K MWCO, Thermo Fisher Scientific, Waltham, MA, USA). Further, 100 μL of 2 M Na_2_CO_3_ was added to 200 μL Zr-89 (37–185 MBq) solution, along with 300 μL 0.5 M HEPES buffer (pH 7.0), 200 μL Df-Bz-NCS-mAb (100–200 μg), and 500 μL 0.5 M HEPES (pH 7.0) and incubated at room temperature for 1 h. Zr-89 ATPS mAb was purified by gel filtration on a PD-10 column (GE Healthcare Bio-Sciences AB, Uppsala, Sweden) and eluted with PBS (pH 7.4) [23]. After radiosynthesis of Zr-89 ATPS mAb, the conjugate was analyzed for labeling efficiency and in-vitro stability using radio-thin-layer chromatography (radio-TLC) (Bioscan, Eckert & Ziegler Radiopharma Inc. Hopkinton, MA, USA), which was performed on the silica gel impregnated aluminium sheets (Merck, Darmstadt, Germany). As a mobile phase, 0.02 M citrate buffer (pH 5.0) was used. In-vitro stability was measured in triplicates on the 2nd and 7th day, in PBS at 4 °C, in FBS at 37 °C or in human serum at 37 °C.

### 4.2. Cancer Cell Lines

Six cancer cell lines, human breast adenocarcinoma (MDA-MB-231), human follicular thyroid carcinoma (FTC133), human fibrosarcoma (HT1080), human gastric adenocarcinoma (MKN45), human prostate adenocarcinoma (PC3), and human lung adenocarcinoma (A549), were obtained from the Korean Cell Line Bank (Seoul, Korea). All cells were grown in Dulbecco’s modified Eagle medium (high glucose; WelGENE Inc., Daegu, Korea) or RPMI-1640 medium (WelGENE Inc.) supplemented with 10% FBS (WelGENE Inc.) and 1% penicillin/streptomycin at 37 °C and 5% fully humidified CO_2_. 

### 4.3. Western Blotting and Confocal Microscopy for ATPS Expression in Cancer Cell Lines

Cancer cells were evaluated for ATPS expression by Western blotting and confocal microscopy. For Western blot analysis, total and plasma membrane proteins were extracted using Minute™ plasma membrane protein isolation kit (Invent Biotechnologies, Inc., Plymouth, MN, USA) according to the manufacturer’s instructions. Samples (10 µg) were loaded on a 12% polyacrylamide gel and transferred to nitrocellulose membranes (Hybond^TM^ ECLTM; Amersham Biosciences, Piscataway, NJ, USA). The membranes were blocked with 5% skim milk and incubated overnight with ATPS mAb at 4 °C, and then with a horseradish peroxidase-linked secondary antibody (1:10,000; Amersham Bioscience). The protein bands were finally visualized with enhanced chemiluminescence reagents (Amersham Bioscience) and exposure to an X-ray film (Agfa^TM^, Mortsel, Belgium). β-actin was used as a loading control. This experiment was repeated twice to confirm the reproducibility. 

For confocal microscopy, cells were grown on chamber slides and stained according to the manufacturer’s instructions. Cells were fixed in 100% methanol for 5 min, permeabilized with 0.1% Triton X-100 for 5 min, and then blocked with 1% bovine serum albumin/10% normal goat serum/0.3 M glycine in 0.1% PBS-Tween for 1 h. The cells were then incubated overnight at 4 °C with ATPS mAb (1 µg/mL). This was followed by incubation at room temperature for 1 h with the secondary antibody at 0.5 µg/mL. Nuclear DNA was labeled with propidium iodide.

### 4.4. Cellular Uptake and Saturation Binding Assay

Cellular uptake of Zr-89 ATPS mAb was measured in MDA-MB-231 and PC3 cells as described previously [24]. In brief, 5 × 10^5^ cells were seeded per well in 12-well plates and cultured overnight. Upon attachment, 37 kBq Zr-89 ATPS mAb was added to freshly replaced culture media, and the cells were then incubated for 1, 2, or 4 h at 37 °C and 5% CO_2_. The cells were washed twice with cold PBS and harvested with 0.1 N NaOH. Radioactivity of the cells was measured using a gamma counter (Shinjin Medics Inc., Goyang, Korea) and normalized to the cell protein content. As controls, the cellular uptake of free Zr-89 and Zr-89 IgG was also measured.

For the saturation binding assay, 1 × 10^5^ cells were seeded per well in 96-well plates [25]. Upon attachment, the cells were grouped into two sets: total and non-specific binding sets. Increasing concentrations (0.1, 0.5, 1, 5, and 10 nM) of Zr-89 ATPS mAb were added to each well and incubated for 4 h. For the non-specific binding set, 200 nM unlabeled ATPS mAb was also added. The cells were washed twice with ice-cold PBS and then harvested with 0.1 N NaOH. Lysates were measured using a gamma counter for radioactivity. Specific binding was calculated by subtracting non-specific binding from total binding. Plots were drawn to calculate K_d_ and B_max_ using Prism ver. 8.0, (Graphpad Software Inc., San Diego, CA, USA).

### 4.5. Xenograft Tumor Model and Biodistribution Analysis

Animal experiments were performed according to protocols approved by the Care of Experimental Animals Committee (IACUC No. 2018-0018, approved date—18 June 2018). To induce xenografts, 1 × 10^7^ MDA-MB-231 or PC3 cells in phenol red-free Matrigel (BD Biosciences, Franklin Lakes, NJ, USA) were injected subcutaneously into the right flank of 6-week-old female or male Balb/c nude mice (Charles River Laboratories Japan, Inc., Yokohama, Japan) and grown for 2 weeks. For biodistribution analysis, 1.85 MBq Zr-89 ATPS mAb was administered intravenously (*n* = 5 for each time point), and then at 4, 24, and 48 h after injection, the mice were anesthetized, sacrificed, and dissected. The amount of radioactivity in the blood, heart, lungs, liver, spleen, stomach, kidneys, intestine, muscle, and tumor was measured using a gamma counter and the percentage of the injected dose per gram tissue was calculated. For the inhibition study (*n* = 3), 60 μg cold ATPS mAb was co-injected with 1.85 MBq (2.4 μg) Zr-89 ATPS mAb in mice bearing MDA-MB-231 tumors and then organs were removed at 24 h after injection. As a control, wild-type mice were injected with 1.85 MBq Zr-89 oxalate, an unlabeled form of Zr-89, and then organs were removed at 24 h after injection.

### 4.6. PET Imaging in Tumor-Bearing Mice

For PET imaging, MDA-MB-231 and PC-3 tumor-bearing mice (*n* = 3, respectively) were administered intravenously with 7.4 MBq Zr-89 ATPS mAb. At 4, 24, 48, and 96 h after administration, tumor images of the mice were obtained using a high-resolution eXplore Vista PET scanner (Sedecal, Spain). Approximately 5 min prior to recording PET images, the mice were anesthetized by inhalation of a 3–4% isoflurane/oxygen gas mixture and placed on the scanner bed in the prone position. List-mode data were acquired for 10 min per scan using a γ-ray energy window of 250–700 keV. Data were reconstructed using a two-dimensional ordered-subset expectation maximum algorithm. The tumor uptake was quantified by SUVmax (g/mL) of the voxel of interests on 24, 48 and 96 h images using a software (AMIDE, SourceForge, San Francisco, CA, USA). 

### 4.7. Confocal Microscopy for Tumor Angiogenesis

MDA-MB-231 and PC-3 tumors removed after PET imaging were used for confocal microscopy. The slides were incubated in a dry oven at 60 °C for 1 h and then deparaffinized and rehydrated by xylene and absolute ethanol thrice. After washing with distilled water, the slides were treated by microwave for 5 min for antigen retrieval thrice. After rinsing with distilled water, peroxidase was blocked using 3% hydrogen peroxide (in methanol) for 10 min. The slides were washed with distilled water and PBS-Tween (0.5%). Tissues were incubated overnight at 4 °C with anti-CD31 antibody (1:50, Abcam, ab28364) and then incubated with the secondary antibody (1:200, Alexa Fluor^®^ 488, A-11034, Thermo Fisher Scientific). Nuclear DNA was labeled with 4′,6-diamidino-2-phenylindole.

The expression of ATPS on the endothelial cells was further evaluated using the slides of MDA-MB-231 xenografts and ATPS mAb under the same protocol. 

### 4.8. Statistical Analysis

All data are presented as mean ± standard error. The statistical comparison of cellular or tumor uptake was evaluated by Student’s *t*-test, and the difference was considered significant at *p* < 0.05.

## Supplementary Materials

The supplementary materials are available online at https://www.mdpi.com/1422-0067/20/16/3928/s1.

## Abbreviations

VEGF	Vascular endothelial growth factor
ATPS	Adenosine Triphosphate Synthase
PET	Positron emission tomography
FDG	Fluorodeoxyglucose
mAb	Monoclonal antibody
Her2	Human epidermal growth factor receptor 2
PSMA	Prostate-Specific Membrane Antigen
PBS	Phosphate buffered saline
FBS	Fetal bovine serum
ID/g	injected dose/gram tissue
Df-Bz-NCS	p-isothiocyanatobenzyl-desferrioxamine
SUVmax	Maximum standardized uptake value
