# Supplementary figures and images for: Zr-89 Immuno-PET Targeting Ectopic ATP Synthase Enables In-Vivo Imaging of Tumor Angiogenesis
Source: Int J Mol Sci. 2019 Aug 13;20(16):3928. doi: 10.3390/ijms20163928 (PMC6720485; doi:10.3390/ijms20163928)

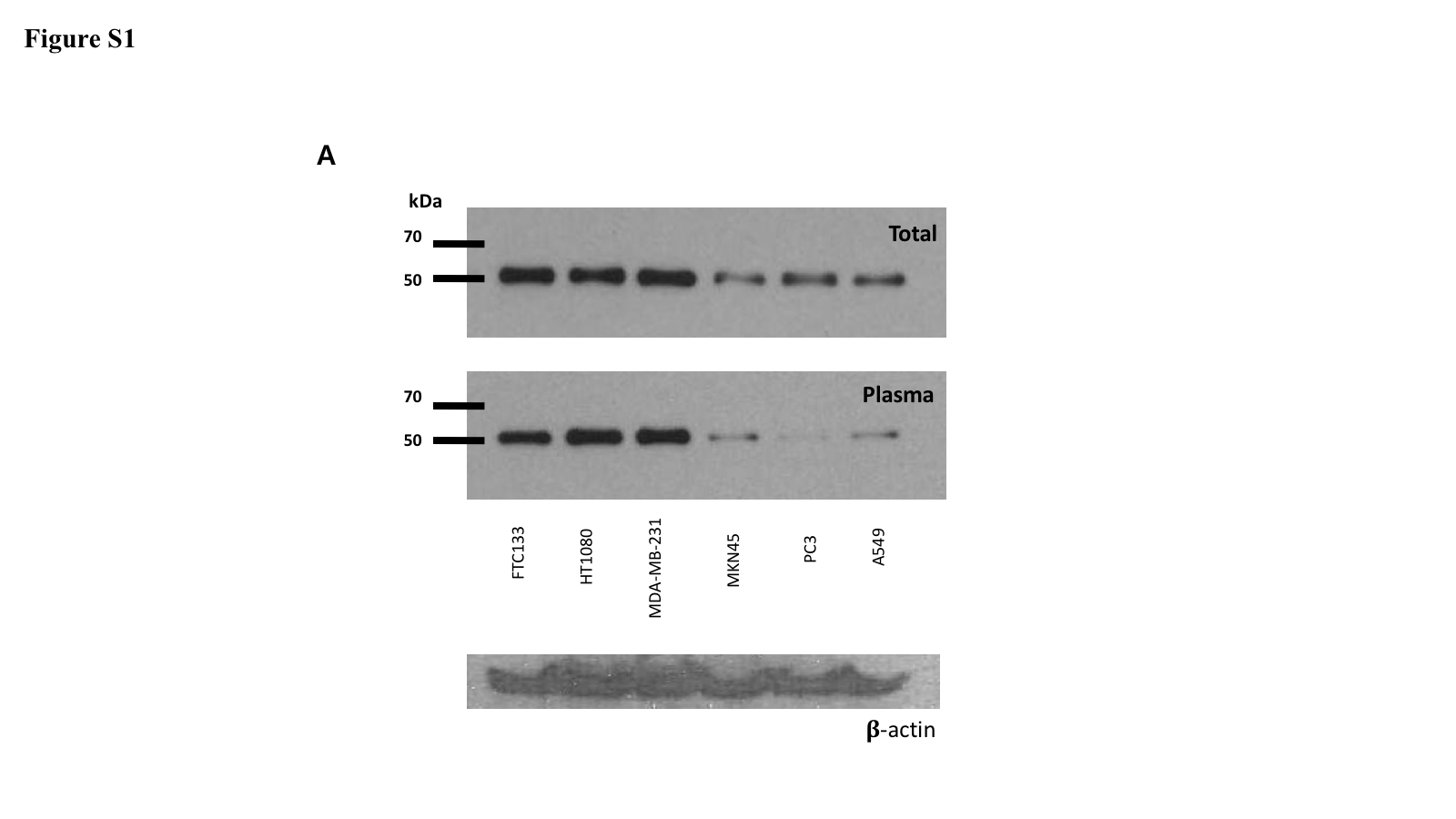

Supplement: Supplementary file 1 [file ijms-20-03928-s001.zip › Fig S1A.tiff]

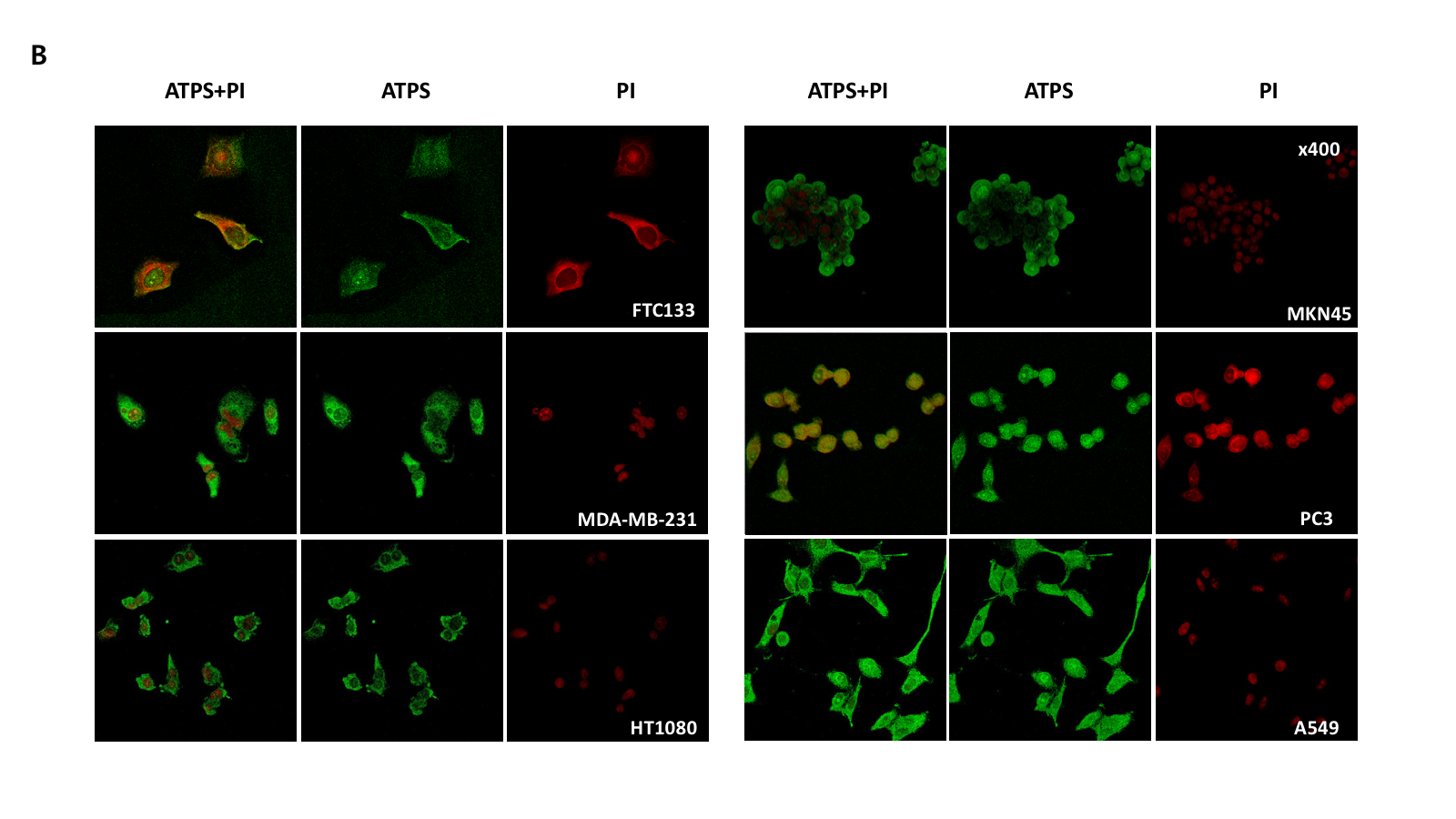

Supplement: Supplementary file 1 [file ijms-20-03928-s001.zip › Fig S1B.tiff]
